# Single Best Answer Questions as a Teaching Tool in Medical Education: An International Mixed-Method Comparative Evaluation

**DOI:** 10.7759/cureus.69745

**Published:** 2024-09-19

**Authors:** Moemen Zegugu, Saif Abbas Chatoo, Anam Choudhry, Huria Metezai, Danyal Usman, Mohammad Kamal, Abdullah El Badawey

**Affiliations:** 1 Internal Medicine, Swansea University Health Board, Swansea, GBR; 2 Internal Medicine, Royal Free London NHS Trust, London, GBR; 3 Internal Medicine, Oxford University Hospitals NHS Foundation Trust, Oxford, GBR; 4 Internal Medicine, Luton and Dunstable NHS Trust, Bedfordshire, GBR; 5 Internal Medicine, Cardiff and Vale University Health Board, Cardiff, GBR; 6 Internal Medicine, Sandwell and West Birmingham NHS Trust, Birmingham, GBR; 7 Internal Medicine, University Hospitals Birmingham NHS Foundation Trust, Birmingham, GBR

**Keywords:** innovative teaching strategies, lecture teaching, medical education, single best answer, virtual

## Abstract

Introduction

Single Best Answer questions are an established assessment format in medical education, however, their use as a teaching tool is underexplored in the literature. We aimed to assess the effectiveness and impact of implementing Single Best Answer (SBA) questions into lecture teaching, compared to standard lectures.

Methods

This evaluation utilised a mixed-method retrospective approach, combining quantitative and qualitative analysis of routine teaching feedback. Over a 2-year period, 10 standard teaching sessions were initially conducted, followed by the development of 43 SBA teaching sessions aimed at improving teaching delivery. Students completed anonymised questionnaires voluntarily after each teaching session (n=3,814 in teaching with SBAs; n=868 in teaching without SBAs). Quantitative data was compared using Welch’s t-test. Statistical analysis was completed using the SPSS version 26.0 software (IBM Corp., Armonk, USA), with *p *< 0.05 considered statistically significant.

Results

The mean perceived confidence increase in topics before and after teaching was significantly higher with the SBA lecture compared to the standard lecture teaching group (1.32 ± 0.14, 1.07 ± 0.12 respectively; *p* < 0.001). Engagement levels were significantly higher in the SBA lecture compared to the standard lecture group (4.55 ± 0.12, 4.21 ± 0.15 respectively; *p* < 0.001). Qualitative data supported these results.

Conclusion

Single Best Answer question use significantly improved student perceived learning outcomes and engagement, indicating its higher efficacy as a teaching tool in our educational programme. This evaluation highlights the potential use of SBA questions to enhance learning in medical education, further studies and testing methods are required to support its wider generalisability.

## Introduction

Medical education is rapidly evolving, driven by digitalisation, and an exponential increase in online teaching platforms which offer globally accessible content. Traditional didactic teaching methods have given way to more dynamic approaches. Some of this can be attributed to the COVID-19 pandemic, when many medical schools shifted to a hybrid model for lecture based teaching, due to official restrictions [1-3]. Students turned to online platforms to supplement learning and to maintain “connectedness” with their peers [4]. A systematic review by Abdull Mutalib et al. found that 72% of studies stated that online learning improved academic performance, but the data was insufficient when comparing teaching styles [5].

As students must navigate a huge number of unvetted resources, a challenge can be to find the most engaging method. Psychological studies show that ‘engagement’ is multidimensional, but a strong predictor of educational outcomes. Students with higher behavioural and cognitive engagement attain higher grades and aspire to higher education [6]. Self directed learning strategies which include taking the initiative to attend extra online teaching, are a timetabled expection for medical students in the United Kingdom, which alongside behavioural and cognitive engagement improves performance [7]. Canton et al. established through timestamp comparisons that online learning allows students to ask more questions at higher complexity after in-person learning environments [8].

Currently, available literature focuses on the success of collaborative learning and peer-assisted learning but fails to delve into the content and format of delivery [9]. This is arguably the way to improve lacking “techno-pedagogical” skills which 85% of the faculty and 88.3% of the students polled in one cross-sectional study, considered a key to engagement [10].

This study delves into the efficacy of Single Best Answer (SBA) questions as a teaching tool in lectures compared with standard lecture teaching. SBA questions are an established assessment format, however their use as a teaching tool is not explored in the literature. The SBA method is characterised by the presentation of a focused question at the start of a subtopic followed by interactive polling. Five options (i.e. Four distractors) are the most common format, and the format used in our teaching [11]. It offers a departure from conventional didactic styles by fostering active participation and discussion. While the utilisation of SBAs in contemporary medical examinations is widespread and suggestions on writing them exist in grey and published literature [12]. Attitudes of the target audience remain underexplored. 

The SBA method was developed from the ‘True/False’ Multiple Choice Question method (MCQ). It was reported by Tan et al. to be more reliable when considering the introduction of the technique in 2006 by the Royal College of Radiologists (RCR) as its sole written test of knowledge for the Final Fellowship Examination in Clinical Oncology [13]. Also, it is worth noting that the United Kingdom Medical Licensing Assessment (UKMLA), which solely uses SBAs, was implemented from 2024 by the General Medical Council (GMC) as the standardised final examination for medical students to join the medical register [14]. 

This project aimed to improve teaching delivery in our educational programme by developing and evaluating the effectiveness of SBA questions as a teaching tool in medical education.

## Materials and methods

Study design

A retrospective analysis of routinely collected feedback data was completed, incorporating both quantitative and qualitative data to draw comparisons. Over a two-year period, a total of 53 educational sessions were conducted through MedAll (Medicinall Limited, Belfast, Norther Ireland) and Zoom (Zoom Inc., San Jose, USA) online platforms as part of an award-winning educational program based in the United Kingdom. Forty-three sessions used the SBA lecture format, while 10 sessions used the standard lecture format. The first 10 sessions were in standard lecture style, but after receiving feedback the program developed and transitioned to the SBA teaching method for the remaining 43 sessions.

The SBA teaching format incorporates at least 7-8 Single Best Answer questions pertinent to the subtopic evenly distributed in the lecture, followed by live polling after each question to gauge initial student understanding and foster engagement. This is followed by a detailed discussion of the SBA question, which clarifies the correct answer, addresses common misconceptions, and links the question to broader concepts. The teaching then proceeds with a focused lecture on the subtopic, integrating insights from the SBA question to enhance relevance and application. This format is highly interactive, offering immediate feedback and directly tying content to practical scenarios. While the standard lecture format involves the instructor delivering content through a traditional presentation of slides or visual aids, with some interactive elements and generally passive student participation. This approach provides a broad overview of the material without the immediate application of specific questions. Instructors were of the same level for all teaching sessions. Duration of all teaching sessions were set at 90 minutes.

Both formats covered equivalent medical school final content for fourth and fifth year students across specialties like Internal Medicine, General Surgery, and Emergency Medicine. Course content was based on United Kingdom National Institute for Health and Care Excellence (NICE) Guidance. The SBA format emphasises active learning and contextual relevance, while the standard lecture format is more traditional and lecture-driven. At the end of each teaching session, students complete a feedback questionnaire. The programme ran twice a year over two years covering the same curriculum for medical student finals. 

Participants

The study included medical students from around the globe, with a predominant representation from the United Kingdom. The number of participants who attended the teaching sessions and voluntarily completed the anonymous feedback response questionnaire during the study period determined the sample size, rather than it being predetermined. A total of n=4,682 questionnaire responses were collected. The selection criteria were medical students internationally in their fourth or fifth year of study who voluntarily attended our free teaching programme to help prepare for medical school finals. 

Effectiveness assessment

A structured questionnaire was designed to measure the effectiveness of teaching sessions, incorporating a modified Likert rating scale ranging from 1 to 5. Four self-evaluation items were used to gauge students’ perceived confidence in topics taught, levels of engagement, format interest, and content helpfulness. A rating of 1 being the lowest of each self-evaluation item and 5 being the highest, with a guided heading according to each questionnaire item (for example "least engaging" and "most engaging"). A qualitative feedback section allowed students to express their opinions through short answer texts providing more detailed and nuanced perspectives.

Statistical analysis

Statistical analyses were con(ducted using SPSS version 26.0 software (IBM Corp., Armonk, USA). Quantitative variables were presented as Mean ± Standard Deviation (SD). Welch’s t-test was used to compare quantitative data between the groups. A significance level of p < 0.05 was set as an indicator of statistical significance. Questionnaire reliability and validity were tested using Cronbach’s alpha measurement. Qualitative data underwent a manual thematic content analysis and coding completed by two investigators independently.

Ethical considerations

The Swansea University Joint Study Review Committee (JSRC) reviewed and approved this project classifying it as a ‘non-research’ evaluation, thus not requiring Research and Development (R&D) and NHS Research Ethics Committee (REC) approval. General Data Protection Regulation (GDPR) notices were flagged to survey participants. Routinely collected feedback was anonymous and voluntary with participants having informed consent prior to project commencement. UK NHS best practice guidelines in ethics and governance were followed.

## Results

Demographic of participants

A total of 3814 participants completed the questionnaire for the SBA lecture sessions, while 868 participants completed the questionnaire for the standard lecture teaching session. The SBA group consisted of 2441 females (64%) and 1373 males (36%). The standard lecture group consisted of 512 females (59%) and 356 males (41%). Among the medical students who completed the questionnaire, 3657 were from the UK (78%), 667 were from Europe (14%), 231 were from Asia (5%), and 127 were from Africa (3%).

As shown in Table 1, the demographic by region of each teaching group is displayed. The most prominent region medical students attended teaching sessions from was the United Kingdom, with international representation seen.

**Table 1:** Demographic Characteristics SBA: Single Best Answer

Demographic characteristics	SBA Teaching Group (N%)	Traditional Lecture Group (N%)
Male	1373 (36)	356 (41)
Female	2441 (64)	512 (59)
Age 18-24	1068(28)	208 (24)
Age 25-35	1450 (38)	356 (41)
Age > 35	1296 (34)	304 (35)
United Kingdom	2937 (77)	720 (83)
Europe	572 (15)	95 (11)
Asia	191 (5)	40 (4.5)
Africa	114 (3)	13 (1.5)
Fourth Year Medical Student	1602 (42)	400 (46)
Fifth Year Medical Student	2212 (58)	468 (54)

Quantitative analysis

In the SBA group, the mean perceived confidence in the topics taught before and after teaching were 2.82 ± 0.18 and 4.15 ± 0.13, respectively. While in the standard lecture group, the mean perceived confidence in the topics taught before and after were 3.07 ± 0.11 and 4.14 ± 0.18 respectively. The mean perceived confidence increases in topics taught were higher in the SBA group versus the standard lecture group, 1.32 ± 0.14 and 1.07 ± 0.12 respectively, this difference was found to be statistically significant (p < 0.001).

Quantitative results

As depicted in Table 2, students’ ratings were superior in the SBA lecture group versus the standard lecture group. This was statistically significant in three out of four self-evaluation items. Cronbach’s Alpha Coefficient was 0.825, indicating a high level of internal consistency and reliability among our self-evaluation items.

**Table 2:** Self-Evaluation Item Ratings After Completion of Teaching Sessions SBA: Single Best Answer

Means (Rating Scale 1-5)	SBA Lecture Group	Standard Lecture Group	P Value (Welch's t-test)
Perceived Confidence Increase	1.32 ± 0.14	1.07 ± 0.12	p < 0.001
Engagement	4.55 ± 0.12	4.21 ± 0.15	p < 0.001
Format interest	4.63 ± 0.10	4.53 ± 0.15	p < 0.05
Content Helpfulness	4.66 ± 0.11	4.59 ± 0.13	p > 0.05

Qualitative analysis

Qualitative questionnaire feedback underwent thematic analysis from both the SBA and standard lecture groups. Below, we outline three themes and patterns that emerged from the collected data. Each feedback example was taken from a separate participant and coded according to the theme it belonged to.

General Perceptions of Learning Theme

Students expressed positive perceptions of learning and reported an improvement in their understanding of the topics covered in both teaching methods. In the SBA teaching group, students highlighted that SBAs provided valuable practice and were felt to improve their understanding. 

Code 1: Overall improvement in understanding of topics.

SBA Lecture Group:

"The lecture provided very clear explanations and an excellent range of questions for better understanding."

“Great revision! There were very clear explanations of management which were really helpful for final year and my understanding. Questions and summary slides were very helpful.”

Standard Lecture Group:

"The lecture was good and helped me gain a better understanding of data interpretation."

"I feel like I learned a lot from the lecture and have a clearer understanding of the topic now."

Code 2: Perceived effectiveness of teaching methods in conveying information

SBA Lecture Group:

“The lecture was very well executed, and the subject matter was presented in a great way.”

"The interaction during the session was good, and the SBAs provided valuable practice for better understanding."

Standard Lecture Group:

"The lecture was informative, the slides contained lots of detailed information."

"The presenter was very knowledgeable and had thorough explanations."

Engagement and Interactivity Theme

The SBA group expressed higher engagement and strongly positive attitudes towards polling and question discussions. While the traditional lecture group also showed positive attitudes, they indicated a greater preference for interactivity, suggesting potentially lower engagement compared to the SBA group.

Code 1: Increased participation and engagement during sessions.

SBA Lecture Group:

“I really liked the SBAs and having the opportunity to attempt to answer in the poll - really engaging!!”

"I really appreciated the SBA questions and the detailed explanations provided afterwards, which enhanced my learning experience."

Standard Lecture Group:

"The Q&A session at the end was useful, it would be better to have more of those."

"I found using the chat function to be really useful to ask questions."

Code 2: Positive feedback on interactive elements like live polling and discussions

SBA Lecture Group:

“I like the use of SBAs throughout to keep everyone engaged”.

“The scenarios and polls were very good and the explanations afterwards were very helpful, the engagement in chat and answering of questions was also extremely helpful”

Standard Lecture Group:

"The presenter was great, and I felt comfortable to ask questions."

"The questions at the end were useful to consolidate my knowledge."

Content Quality and Format Theme

Both teaching groups found content quality comprehensive and highly relevant to their medical curriculum. Suggestions in improvement for SBA teaching involved allowing more time for polling and reading of the question. The traditional teaching group reported that slides can be long and didactic in nature, which may lead to increased disengagement. Students felt the teaching material was well presented and high yield information was displayed clearly in both groups. 

Code 1: Clarity and organization of teaching materials

SBA Lecture Group:

“I really liked the question format! It's a first to being able to answer questions in real time and get an explanation afterwards”.

"The teaching materials were very well organized, with clear sections for each topic covered."

Standard Lecture Group:

"The content covered in the lecture was well-organized and easy to follow."

"The lecture slides were well structured and comprehensive!”

Code 2: Relevance of content to curriculum and learning objectives

SBA Lecture Group:

“Great summaries and SBAs relevant to exams and OSCEs, engaging and well explained”.

“Q and A really helps brainstorm and encourages learning from mistakes just like an exam situation, except that we don't get penalized for the wrong answers which is the best thing.”

Standard Lecture Group:

"High yield topics, very relevant to my exams”

"Great consolidation to what we learn in lectures.”

Code 3: Suggestions for improvement in content delivery or presentation style

SBA Lecture Group:

“Could you allow some time to read the question before the poll is brought up please?”

“It would be better if the doctor could recall the concepts first before question session”.

“Including SBA-options explanations on screen”.

Standard Lecture Group:

“More Q&A and opportunities for discussion would be great!”

"I found the teaching super helpful, but I think it could have been a little more engaging, slides were long and overwhelming at times."

## Discussion

The findings indicated significantly higher levels of mean perceived topic confidence, engagement, and format interest in the material taught for the SBA lecture group compared to the standard lecture group. The quantitative data in tandem with more positive responses in the SBA qualitative data suggested clarity of subject material due to engagement compared with the standard teaching model. 

The difference in ‘helpfulness of content’ between the SBA and standard sessions was not found to be statistically significant. This does not impact our hypothesis but rather supports that the quality of information taught was consistent among both groups. In fact, it improves the validity of results by removing content quality as a variable.

By considering complementary auditory and visual channel theory, we aimed to uphold consistency of the slide design, length of each session (maximum 1.5 hours) and ‘summary cheat sheets’ [15]. Teachers were of similar academic experience, and all presentations were proofread by two reviewers. It is notable that positive feedback on didactic sessions centred around the teacher’s engaging delivery style, whereas SBA teaching feedback commented on the method itself. SBAs by nature require the application of knowledge. “De-emphasizing” the teacher stops information overload and puts responsibility on the learner [16]. Educational cognitive theory backs the organisation of information in memory via schemas and themes, and a departure from rote-learning facts to memory, which would not prepare the learner for SBA exams [17].

Retaining engagement in didactic teaching can be challenging. Although evidence for the 10-minute attention span of learners is somewhat contested and variable [18,19]. We suggest that SBAs manage to retain interest by dividing up lecturer speech. Students are willing to respond because they hold value in mimicked exam questions and the ease and anonymity of aggregated polls. 

Furthermore, immediate feedback and explanation of scenarios is the forte of the SBA method [20]. This was reflected in our qualitative analysis. Responses emphasised that answering questions in real time followed by discussion, simulated a practice exam scenario without penalty which is a positive experience. There was one comment suggesting that SBAs at the end of topics would be preferred. However, we suggest that it would not give students a real sense of where their prior knowledge was. Therefore, the metric of “before” and “after” used to gauge confidence increase would be falsely elevated without a benchmark for each topic. The initial question acts as a scaffold for the topic, which is a recognised education method [21].

Studies in the literature comparing very short answer (VSA) questions versus SBAs have found that VSAs by nature, are more challenging, therefore testing ‘nascent physician ability’ rather than exam passing [22,23]. However, our study aimed to consider SBAs as a teaching technique to prepare students for exams, rather than commenting on its use as an examination modality. It is surprising that the literature on employing SBAs as a teaching method is relatively sparse. Considering the official use of SBAs in standardised national summative assessments such as: the United Kingdom Medical Licensing Assessment (UKMLA), the United States Medical Licensing Examination (USMLE), the Medical Council of Canada Qualifying Examination (MCCQE), and the Membership of the Royal Colleges of Physicians (MRCP) examinations [14,24,25].

Our study provided valuable insights into the effectiveness of SBA incorporated lectures in comparison to traditional lecture teaching in virtual medical education, highlighting its potential to enhance student engagement and learning outcomes.

Strengths and limitations

The data set collected was large and robust. The mixed-method approach allowed a holistic insight into the effectiveness of each teaching method and improved the richness of the data. Additionally, appropriate statistical calculations were employed. Welch's t-test is suitable for unequal sample sizes and variance, and accounts for slight deviations from normality within the data set when given a large sample size like ours.

One limitation in our study is an unequal number of teaching sessions between study groups for an improved comparison. However, due to feedback after the first 10 sessions, the SBA lectures were developed in order to improve teaching delivery

## Conclusions

In conclusion, employing SBA questions as a teaching tool significantly improved student confidence in topics, and fostered higher levels of engagement when compared to stand alone lecture teaching. This suggests that SBA-incorporated lectures were more effective and lead to better learning outcomes for students in our teaching programme. Additional prospective research studies and testing methods using Single Best Answer questions are needed to support the generalisability of this teaching format.
